# Low responsiveness of peripheral lymphocytes in extraparenchymal neurocysticercosis

**DOI:** 10.1371/journal.pntd.0011386

**Published:** 2023-06-01

**Authors:** Matthew L. Romo, Rocio Osorio, Andrea Toledo, Roger Carrillo-Mezo, Ricardo Valdez, Marta C. Romano, Edda Sciutto, Gladis Fragoso, Agnès Fleury

**Affiliations:** 1 Department of Epidemiology & Biostatistics, CUNY Graduate School of Public Health and Health Policy, City University of New York, New York, United States of America; 2 Unidad de Neuro inflamación, Departamento de Medicina Genómica y Toxicología ambiental, Instituto de Investigación Biomédicas, Universidad Nacional Autónoma de México (UNAM) / Instituto Nacional de Neurología y Neurocirugía, Ciudad de México, México; 3 Neurocysticercosis Clinic, Instituto Nacional de Neurología y Neurocirugía, Ciudad de México, México; 4 División de Investigación, Facultad de Medicina, Universidad Nacional Autónoma de México (UNAM), Ciudad de México, México; 5 Neuroradiology Department, Instituto Nacional de Neurología y Neurocirugía, Ciudad de México, México; 6 CINVESTAV, Departamento de Fisiología, Biofísica y Neurociencias, México; 7 Departamento de Inmunología, Instituto de Investigación Biomédicas, Universidad Nacional Autónoma de México (UNAM), Ciudad de México, México; Universidad de la República Uruguay: Universidad de la Republica Uruguay, URUGUAY

## Abstract

**Background:**

The morbidity and mortality of extraparenchymal neurocysticercosis (EP-NC) remain high and effectiveness of current medical treatment is suboptimal. Various factors have been implicated in the severity of EP-NC and in the poor response to treatment, but the possible role of host immune and endocrine systems has not yet been examined thoroughly.

**Methodology/Principal findings:**

42 participants with EP-NC before receiving standard treatment and 25 healthy controls were included in the study. Treatment response was assessed by comparing pre/post treatment parasite volumes from 3D MRI. Prior to treatment among participants with EP-NC, specific stimulation induced an increased specific proliferative response accompanied by a significant increase in IL-4, NK, NKT, Bregs and Tregs cells, whereas in healthy controls, specific stimulation induced a significant increase in IL-1β, IL-5, CCL5, IL-6, TNF-α, NK and Bregs cells. Significant differences between participants with EP-NC and healthy controls in the specific inflammatory response were observed. Participants with EP-NC prior to treatment had significantly weaker responses of proinflammatory cytokines (IL-6, TNF-α) and NK cells, and stronger IL-4 response. Anthelmintic treatment did not promote significant peripheral immunological changes at any time, although inflammation was sustained in the cerebrospinal fluid. Serum estradiol concentration significantly decreased after anthelmintic treatment among males, and cortisol correlated negatively with IL-6 and positively with IFN-γ levels. No pre-treatment immunologic or endocrinologic parameters were significantly associated with response to treatment.

**Conclusion/Significance:**

Prior to anthelmintic treatment, EP-NC was characterized by low lymphocyte reactivity accompanied by a regulatory response, which may be involved in the lack of peripheral immunological changes during and after treatment, although a central inflammatory response was present. This weak specific peripheral response could favor the chronicity of the infection and the poor response to treatment. Our findings highlight the need for new anti-inflammatory treatment focused on the central nervous system with less systemic immunosuppressive effects.

## Author summary

Neurocysticercosis is a central nervous system infection with the helminth *Taenia solium* in its larval stage. It is a marker of poverty and remains endemic in countries of Latin America, Africa, and Asia. The severity of neurocysticercosis and the prognosis of patients depend on the location of the parasites. Extraparenchymal infection involving the subarachnoid and/or ventricular space is associated with high morbidity and mortality and a suboptimal response to current anthelminthic treatment regimens. We examined peripheral immunological parameters and hormones of 42 participants with extraparenchymal neurocysticercosis before and after treatment, and their associations with subsequent treatment response. Prior to treatment, compared with healthy controls, there was low lymphocyte reactivity with a regulatory response, which may explain the lack of peripheral immunological changes during and after treatment. This weak specific peripheral response could favor the chronicity of the infection and the poor response to treatment and should be considered in research to improve patient outcomes.

## Introduction

Extraparenchymal neurocysticercosis (EP-NC) is the less frequent but most severe form of NC [1,2]. Different factors contribute to its severity. First, the symptoms of EP-NC are more serious than parenchymal NC and frequently require emergency intervention. In fact, potentially fatal intracranial hypertension is the most frequent manifestation, often requiring neurosurgical management, mainly by the placement of a ventriculoperitoneal shunt. Second, anthelmintic treatment is less effective than when parasites are only located in the parenchyma. However, EP-NC is a highly heterogenous disease; some patients respond well to a single cycle of anthelmintic treatment while others require multiple treatment cycles [3].

The safety and efficacy of anthelmintic treatment for EP-NC has not been adequately evaluated in randomized trials, but research has been conducted by our group to understand the factors involved in the heterogeneity of treatment response for EP-NC. Specifically, we found that increased plasma levels of albendazole sulfoxide, decreased pretreatment parasite volume, and fewer cysts were significantly associated with improved treatment response [3].

Evidence also suggests that the host immune and endocrine systems are involved in treatment response [4]. Regarding the immune system, in a cross-sectional study, the expression of proinflammatory genes was associated with a better response to treatment [5]. Also, in the experimental murine model with *Taenia crassiceps*, a parasite that closely resembles *T*. *solium*, the administration of corticosteroids concomitantly with albendazole reduced cyst resolution [6]. Based on these findings, we proposed that a certain degree of host inflammation is additive to the effect of anthelmintic drugs and might result in more effective destruction of the parasite and thus, better treatment response [7].

Regarding the endocrine system, patients with NC have substantial hormonal changes compared with individuals without NC and these changes are associated with disease severity. For example, in a cross-sectional study, serum progesterone and androstenedione levels in females and testosterone levels in males were significantly lower when comparing clinically severe NC to clinically non-severe NC, while follicle-stimulating hormone among males was significantly higher in the severe group [8]. Considering the relevance of the immune-endocrine interactions in the outcomes of different neurological diseases [9], it is possible that the endocrine system could also modulate treatment response in EP-NC.

Understanding the role of immune and endocrine systems in this severe presentation of NC and their effects on patient outcomes after treatment is essential, as this knowledge might allow us to improve upon the existing treatment strategies.

## Methods

### Ethics statement

This study complied with all Mexican and international laws and regulations for ethical clinical research practice, and the study protocol was approved by the ethical committee of Instituto Nacional de Neurología y Neurocirugía (63/14). All participants provided written informed consent for their neurological and radiological information and blood samples to be used for research purposes.

We conducted a longitudinal study from June 2014 to April 2019 in which all patients with a confirmed diagnosis of vesicular (viable) EP-NC requiring the administration of anthelmintic treatment and attending the Instituto Nacional de Neurología y Neurocirugía (INNN) in Mexico City were invited to participate. We also included a control group of healthy subjects matched by age and sex to compare immunological findings. Participation was voluntary. Most of the participants in this study (31/42) were also included in a previous one in which we assessed the role of disease and host characteristics, as well as plasma albendazole sulfoxide levels in treatment response [3]. Some aspects of the methods are similar and are summarized below.

### Participant enrollment and treatment

#### Participants with EP-NC

The inclusion criteria were to have a diagnosis of vesicular EP-NC based on validated diagnostic criteria [10], to accept the 15-day hospitalization period and standard medical treatment, to not have been treated with anthelmintic drugs (albendazole, praziquantel, or ivermectin) and corticosteroids in the past 6 months, to agree to attend follow-up visits at 1 and 6 months after treatment, and to agree to give blood samples before treatment and during the two follow-up visits. Exclusion criteria were uncontrolled metabolic diseases, neoplastic diseases, other infectious or neurological diseases, and pregnancy.

The treatment protocol, used at INNN for more than 10 years, consisted of oral albendazole (30 mg/kg/day in 2 or 3 divided doses) for 10 days and corticosteroids (intravenous dexamethasone 0.4 mg/kg/day in 3 divided doses for 13 days, followed by oral prednisone 50 mg/day with a taper) [11]. This treatment protocol differs from what is recommended in recent US guidelines, that is, to continue treatment until a resolution of cystic lesions on neuroimaging studies occurs [12]; however, there are no controlled studies that support this recommendation [13]. The main adverse events of this regimen were a reversible increase of liver enzymes and reversible alopecia in fewer than 10% of participants. Standard laboratory tests (complete blood count, liver and kidney function) were within normal limits at baseline, at the end of treatment, and in the follow-up visits. Blood glucose concentration was also within normal limits at baseline among all participants. Some participants developed hyperglycemia during dexamethasone treatment that required the use of antihyperglycemic agents.

#### Healthy controls

Family members of patients attending INNN and staff and students were invited to participate as healthy controls. Exclusion criteria were uncontrolled metabolic diseases, neoplastic diseases, neurological and infectious diseases (including NC), and pregnancy.

### Outcome: Evaluation of treatment response

All participants with EP-NC underwent a neurological evaluation and a cerebral MRI with 3D sequences (FIESTA) before initiating treatment and 6 months after completing treatment [14]. The evaluation of partial parasite volumes (pvol) in each axial level was conducted, and global parasite volume was determined using the following formula: (pvol1+pvol2+....+pvoln)×axial MRI slice thickness. Only volumes of parasites in vesicular stages were considered (that is, with a fluid intensity equal to the cerebrospinal fluid intensity). This determination was made by 2 researchers, a neuroradiologist (R.C.M.) and a neurologist (A.F.), both experts in NC. Treatment response was percent volume reduction between pre- and posttreatment volumes, with a volume reduction of 100% considered complete treatment response and 0% considered no treatment response. Interrater reliability for treatment response was substantial between the 2 reviewers (kappa: 0.65; 95% confidence interval: 0.42–0.88). When there was disagreement, the 2 reviewers discussed the case together to reach a consensus.

### Evaluation of systemic inflammation

A battery of immunological parameters was evaluated before treatment and at 1 and 6 months after treatment. A 20 mL venous blood sample drawn from participants was obtained in tubes coated with ethylenediamine tetra-acetic acid (EDTA) (BD Vacutainer). The samples were kept at room temperature and processed within 2 hours. Peripheral blood mononuclear cells (PMBCs) were isolated in a Lymphoprep gradient.

#### Antigen preparation

*Taenia solium* antigen was obtained from cysticerci as reported previously [15] with modifications. Briefly, skeletal muscle cysticerci of one infected pig from central Mexico were washed with phosphate-buffered saline solution, each cysticercus was punched with a sterile needle and the vesicular fluid was collected. Thereafter, the fluid was filtered under sterile conditions and the total protein concentration was quantified using the kit Pierce BCA protein assay kit (Thermo Scientific). Then samples were aliquoted, labeled, and frozen at -20°C until used. The level of lipopolysaccharide (LPS) in the vesicular fluid was quantified using a commercial kit (Endochrome-k, Charles Rivers Laboratories, Inc., Charleston, USA). According to this kit we found a concentration of 0.05 ± 0.01 ng/mL in the vesicular fluid of 1 mg/mL of protein. This level of LPS is below the value authorized by the US Food and Drug Administration for intravenous injectable drugs.

#### PMBC isolation, cell staining, and *in vitro* stimulation with *T. solium* antigen

As described above, a venous blood sample was obtained in tubes coated with ethylenediamine tetra-acetic acid (EDTA) and diluted 1:1 with 1640-RPMI, layered over 10 ml of Lymphoprep (Axis -Shield, Oslo Norway). PBMCs were washed twice with sterile PBS 1x and suspended in RPMI-1640 supplemented with 10% of human AB serum (donated from the blood bank of INNN Manuel Velasco Suarez, Mexico City), 2 mM L-glutamine, 100 U/mL penicillin, 100 mg/mL streptomycin, 1% non-essential amino acids, and 1% pyruvate (Gibco BRL) and used for cell culture. The proliferative response was measured by staining 10 x10^6^ PBMC with carboxyfluorescein diacetate succinimidyl ester (CFSE, Biolegend Cat. 423801) at 5μM diluted in RPMI-1640 for 5 minutes then washed twice with RPMI-1640 medium supplemented with 10% fetal bovine serum.

To evaluate the specific proliferative response and the induced cytokines, 2 x 10^6^ CFSE stained cells/mL were incubated with 10 μg/mL of *T*. *solium* antigen in supplemented RPMI or only with RPMI at 37°C in a 5% CO2 humidified atmosphere in 24-well flat-bottom culture plates (Costar, Cambridge, MA). After 72 hours of culture, 2 ml of the supernatants of each of the evaluated conditions were harvested and stored at -80°C until use for cytokine quantification. For intracellular cytokine quantification, cells were cultured for 90 hours, and treated with 20 μl of Brefeldin for 6 hours before cell collection. Cells were stained to evaluate the phenotype of proliferating cells by flow cytometry. Lymphocyte proliferation was assessed by the decrease of the cell fluorescence (due to CFSE dilution in each cell cycle) measured also through flow cytometry.

#### Peripheral cells phenotype (flow cytometry)

The immunophenotype of PBMC stimulated with *T*. *solium* vesicular fluid or medium alone was measured using standard phenotyping protocols provided by the manufacturers. The immunophenotype was carried out by flow cytometry in 15 of the 42 participants with EP-NC and in the 25 healthy controls.

For CD4 T cells, cultured cells were stained with anti-CCR7, anti-CD45RA, anti-CD27 and anti-CD28 for 30 minutes at room temperature. Thereafter cells were washed twice with PBS containing 10% fetal bovine serum and finally suspended in PBS containing 2% of paraformaldehyde (PFA). To determine the percentage of proliferating effector cells, CD27+/CD28+ expression was determined within the CCR7-/CD45RA- subset of the total CD4+ T-cell population. The percentage of CCR7+/CD45RA+ (naive) and CCR7+/CD45RA- (central memory) was also analyzed in proliferated T CD4^+^ cells.

For B cells, the cultured cells were stained with anti CD19 mAb for 30 minutes at room temperature, subsequently the cells were washed with PBS containing 10% fetal bovine serum (PBS/10% FBS), then cells were fixed and permeabilized for 18 hours with fix/perm buffer (eBioscience) and stained again with anti IL10 intracellular antibody for another 30 minutes. Finally, the cells were washed with PBS/10% FBS and fixed with PBS containing 2% of PFA.

For Tregs (CD4^+^/CD25^+^/FoxP3^+^), NK (CD3^-^/CD16^+^/CD56^+^), NKT (CD3^+^/CD16^+^/CD56^+^) direct labeling was performed as described above using the respective antibodies.

The percentage of antigen stimulated cells was analyzed, acquiring at least 30,000 target events, by using an Attune NXT (BD Biosciences) and analyzed with the CellQuest software.

The human monoclonal antibodies used for leukocyte phenotyping by flow cytometry were APC/FIRE 750 anti-CD4, PerCP anti-CD45RA, PE anti-CCR7, Alexa Fluor 700 anti-CD27, allophycocyanin (APC) anti-CD28, APC anti-CD25, PE anti-FoxP3, PE anti-CD19, APC anti-CD3; PE anti-CD16; PerCP anti-CD56 were obtained from Biolegend (San Diego, CA, USA). All antibodies were titrated for optimal detection of positive populations prior to use, following the manufacturer’s (BioLegend) recommended concentrations. Appropriate isotype controls were also included.

For cytometric analyses, cells were first selected according to forward and sideways light scattering properties of lymphocytes. From these, the population of total proliferative response was analyzed with CFSE marker, and all the other specific study populations were determined. The strategy of the analysis is depicted in S1 Fig.

#### Cytokine titration (ELISA)

Commercial sandwich enzyme linked immunosorbent assay (ELISA) kits were employed to quantify in supernatant of culture cells with or without *T*. *solium* antigen, the pro- and anti-inflammatory human cytokines IL-1β, IL-4, IL-5, IL-6, IL-17A, CCL5, and INF-γ (all from BioLegend, San Diego, CA, USA), following the supplier’s instructions. Sandwich ELISAs were performed in 96-well, flat-bottom microtiter plates (Nunc-Immuno Plate Maxisorp). The determination of cytokines in the supernatants was carried out in 31 to 42 participants with EP-NC and in the 25 controls included. The detection limits were 3.9 pg/ml for IL-1β and IL-4, 7.8 pg/ml for IL-5 and IL17-A, 15.6 pg/ml for IL-6, CCL5, IFN- γ and TNF-α. All analyses were run in duplicate.

### Evaluation of central inflammation

Lumbar puncture to evaluate cerebrospinal fluid (CSF) cellularity (cells/mm^3^), protein count (mg/dL), and glycorrhachia (mg/dL) was performed in 38 participants with EP-NC (90.5%) before treatment and in 37 participants (88.1%) 6 months after treatment.

### Evaluation of peripheral hormones

Shortly after blood collection, serum was separated and maintained at -20°C until required. The concentration of the following hormones was measured by radioimmunoanalysis (RIA) using ^125^Itracers kits: testosterone (TESTO-CT2, only in men), 17b-estradiol (E2, ESTRCTRIA), prolactin (RIA-gnost PROL), cortisol (CORT-CT2), all from CIS-bio International (Gif sur Yvette, France). The detection limits were: E2, 8 pg/mL; cortisol, 4.6 nmol/L; testosterone, 0.1 nmol/L and prolactin, 5 uU/mL.

### Statistical analysis

We first characterized the sample using descriptive statistics. Pearson’s chi-square tests were used to compare treatment response among participants with EP-NC by prior anthelmintic treatment. Wilcoxon-signed rank tests were used to compare changes in CSF cells, protein, and glucose before and after treatment among participants with EP-NC. To compare participants with EP-NC and healthy controls, a Pearson’s chi-square test was used for sex and a Mann-Whitney U test was used for age.

We computed median level, interquartile range, and minimum-maximum range for each cytokine (IL-1β, IL-17A, CCL5, IL-6, IFN-γ, IL-4, IL-5, TNF-α) and cell phenotype (% Proliferative, % Naïve, % Central memory, % Effector memory, % Bregs, % NKT, % NK, %Tregs) before and after *T*. *solium* antigen stimulation among healthy controls and participants with EP-NC prior to anthelmintic treatment. We conducted complete case analyses, and discrepancies in sample sizes were a result of not being able to conduct peripheral cell phenotyping by flow cytometry for all participants with EP-NC due to funding limitations and insufficient sample quantity for some cytokines. Differences in immunological parameters before and after antigen stimulation (i.e., specific inflammatory responses) were compared using Wilcoxon signed-rank tests. Comparing healthy controls and participants with EP-NC, we used Mann-Whitney U tests to assess between-group differences in parameters before and after antigen stimulation and specific inflammatory responses. Among participants with EP-NC, we used Wilcoxon-signed rank tests to compare changes in specific inflammatory responses between time points (i.e., pre-treatment to 1-month post-treatment, 1-month post-treatment to 6 months post-treatment, and pre-treatment to 6 months post-treatment). Spearman correlation coefficients were computed to examine the correlations between specific inflammatory responses and CSF cells, protein, and glucose, both prior to treatment. We assessed the association of the pre-treatment specific response for each immunological parameter with treatment response (i.e., percent volume reduction of vesicular cysts) using multivariable linear regression models adjusted for age and pre-treatment parasite volume.

For peripheral hormones, we computed median levels and ranges at pre-treatment, 1-month post-treatment, and 6-months post-treatment stratified by sex, with estradiol further stratified by menopausal status for females. We compared changes in peripheral hormone levels between time points using Wilcoxon signed-rank tests. To assess the association of each peripheral hormone with treatment response, we used the same approach as with immunological parameters, but also adjusted for sex. We computed Spearman correlation coefficients to examine associations between pre-treatment peripheral hormones with pre-treatment specific inflammatory responses among participants with EP-NC.

All analyses were conducted using SAS 9.4 (SAS Institute Inc., Cary, NC) and statistical significance was determined by an alpha level of 0.05.

## Results

### Characteristics of the included participants

Overall, 42 participants with EP-NC were included with a median age of 49 years (interquartile range [IQR]: 41–56) and 17 (40.5%) were female. Overall, 30 (71.4%) had cysts located only in the subarachnoid space, 4 (9.5%) had cysts only in the ventricles, and 8 (19.1%) had cysts in both locations. At inclusion, participants’ primary neurological symptom was headache (36 [85.7%]), dizziness (2 [4.8%]), epilepsy (2 [4.8%]), and cognitive impairment (2 [4.8%]). Median pre-treatment volume of vesicular cysts was 3839 mm^3^ (IQR 2132–9120) and median volume at the end of follow-up (i.e., 6 months post-treatment) was 657 mm^3^ (IQR 1–2453). At the end of follow-up, 11 (26.2%) participants had a complete treatment response (100% volume reduction), 25 (59.5%) had volume reduction between 50–99%, and 6 (14.3%) had a volume reduction <50%.

Of the 42 participants with EP-NC, 23 had never received anthelmintic treatment (with corticosteroids) before inclusion. Of the 19 participants who had received prior treatment >6 months before inclusion, 8 had received it 6–12 months before inclusion and the other 11 had received it more than 12 months before inclusion. There was no significant difference in treatment response between participants with and without prior treatment (p = 0.45). When dividing participants according to the time of previous treatment (>12 or 6–12 months prior to inclusion), a significantly improved response was seen in those who had received it >12 months ago compared with those who had received it more recently (p = 0.017) and compared with those who had never received treatment before (p = 0.048).

Among participants with EP-NC, pretreatment CSF showed an inflammatory reaction with a median of 44 cells/mm^3^ (IQR 18–124; normal values <5 cells/mm^3^), median protein 106.0 mg/dL (IQR 57.0–370.0; normal values <45 mg/dL), and median glucose 43.5 mg/dL (IQR 15.0–59.0; normal values 40–85 mg/dL). At 6 months after treatment, median number of cells was 34 cells/mm^3^ (IQR 17–89), median protein was 94.0 mg/dL (IQR 60.0–340.0), and median glucose was 46.0 mg/dL (IQR 30.0–57.0). As reported in our previous paper [3], overall, changes before and after treatment were not statistically significant. When data were stratified by treatment response, the only significant change was the increase in CSF glucose in participants with a complete response (p = 0.031).

Twenty-five healthy controls were included with a median age was of 49 years (IQR 40–51) and 10 (40%) were female. Sex and age between participants with EP-NC and controls were not significantly different (sex p = 0.969 and age p = 0.344).

### Changes in immunological parameters after specific stimulation among participants with EP-NC before treatment and healthy controls

As shown in **Table 1**, prior to stimulation of PBMC by *T*. *solium* antigen, participants with EP-NC had a higher concentration of IL-1β (p = 0.007), IL-4 (p = 0.048), IL-6 (p = 0.038) and a lower concentration of TNF-α (p<0.001) compared with healthy controls. After stimulation, differences for IL-4 (p = 0.031) and TNF-α (p<0.001) comparing participants with EP-NC and healthy controls persisted. Regarding cells, before stimulation healthy controls had a higher percentage of T-regulator cells (p = 0.031), while before and after stimulation participants with EP-NC had significantly higher percentages of effector memory cells (p = 0.041 and p = 0.016, respectively), and healthy controls had significantly higher percentages of naïve (p = 0.007 and p = 0.019, respectively) and NK cells (p = 0.029 and p<0.001, respectively). Stimulation of PBMC by *T*. *solium* antigen induced a statistically significant proliferative response in participants with EP-NC (p<0.001) but not in healthy controls (p = 0.330). In participants with EP-NC, there were significant increases in IL-4 (p = 0.016), B- and Tregs (p = 0.003 and p = 0.022, respectively), NK (p<0.001) and NKT (p = 0.003). In healthy controls, there were significant increases in IL-1β, CCL5, IL-6, TNF-α (p<0.001 for all) and IL-5 (p = 0.004), and in Bregs (p = 0.006) and NK (p<0.001).

**Table 1:** Immunological parameters before and after *Taenia solium* antigen stimulation among healthy controls and participants with EP-NC before treatment.

	Healthy controls (n = 25)			Participants with EP-NC prior to treatment (n = 42)			p-values for comparisons			
	n	Before Ag stimulation, median [IQR] (min-max)	After Ag stimulation, median [IQR] (min-max)	n	Before Ag stimulation, median [IQR] (min-max)	After Ag stimulation, median [IQR] (min-max)	Healthy controls: after vs. before Ag stimulation*	EP-NC: after vs. before Ag stimulation*	Before Ag stimulation: controls vs. EP-NC p-value^†^	After Ag stimulation: controls vs. EP-NC p-value^†^
IL-1β, pg/mL	25	38.2 [0–56.2] (0–131.6)	66.0 [20.4–94.3] (0–130.0)	42	72.4 [8.3–153.3] (0–5492.6)	48.8 [2.2–242.8] (0–9425.0)	**<0.001**	0.372	**0.007**	0.555
IL-17A, pg/mL	25	0 [0–0] (0–2.2)	0 [0–0] (0–6.4)	38	0 [0–0] (0–310.9)	0 [0–0.8] (0–275.1)	0.063	1.000	0.284	0.413
CCL5, pg/mL	25	304.4 [228.7–327.3] (31.4–391.3)	334.6 [257.6–364.3] (184.8–407.9)	35	338.7 [125.0–473.4] (0–713.1)	356.4 [260.6–480.6] (0–1887.0)	**<0.001**	0.156	0.401	0.230
IL-6, pg/mL	25	224.2 [121.1–375.6] (7.2–631.2)	403.6 [163.6–570.5] (0.2–670.9)	40	486.3 [130.1–733.3] (0–50996.3)	311.0 [80.4–631.5] (0–62348.8)	**<0.001**	0.826	**0.038**	0.671
TNF-α, pg/mL	25	2.8 [0.3–5.0] (0–11.7)	5.0 [3.1–8.1] (0–81.8)	34	0 [0–0] (0–1.0)	0 [0–0] (0–142.9)	**<0.001**	0.500	**<0.001**	**<0.001**
IFN-γ, pg/mL	21	0 [0–0] (0–162.1)	0 [0–0] (0–170.5)	36	0 [0–0] (0–438.1)	0 [0–0] (0–688.9)	1.000	0.078	0.150	0.088
IL-4, pg/mL	25	0 [0–0] (0–0)	0 [0–0] (0–0)	41	0 [0–0] (0–13.3)	0 [0–0] (0–139.3)	‡	**0.016**	**0.048**	**0.031**
IL-5, pg/mL	22	0 [0–0.2] (0–1.3)	0.1 [0–0.8] (0–4.1)	31	0 [0–0] (0–38.7)	0 [0–0] (0–17.6)	**0.004**	1.000	0.246	0.108
Proliferative, %	25	6.7 [4.6–9.0] (3.0–28.5)	6.6 [4.6–10.0] (3.0–29.6)	38	11.8 [6.1–14.9] (0.8–41.5)	15.5 [6.2–26.4] (1.4–84.1)	0.330	**<0.001**	**0.018**	**0.013**
Naïve, %	25	59.0 [37.4–78.2] (1.1–97.8)	53.6 [33.6–76.8] (0.6–97.6)	15	13.5 [3.5–50.8] (0.3–70.2)	20.7 [2.6–49.0] (0.1–74.7)	0.223	0.252	**0.007**	**0.019**
Central memory, %	25	31.6 [4.7–41.6] (0.8–81.7)	26.8 [5.4–45.3] (0.6–89.6)	15	38.1 [4.1–57.3] (0–88.9)	39.6 [3.3–60.7] (0–90.1)	0.647	0.455	0.503	0.567
Effector memory, %	25	1.2 [0.3–15.5] (0–96.0)	1.6 [0.4–7.2] (0–95.3)	15	11.5 [1.1–33.4] (0.3–69.3)	12.7 [3.9–30.8] (0.5–71.2)	0.294	0.330	**0.041**	**0.016**
Bregs, %	25	2.5 [0.4–8.2] (0–37.4)	5.2 [0.5–14.0] (0–52.2)	15	3.2 [0.9–5.3] (0.1–5.6)	4.5 [1.1–8.1] (0.1–13.7)	**0.006**	**0.003**	0.967	0.605
NKT, %	25	11.1 [4.1–21.4] (0.5–77.7)	9.6 [5.0–20.8] (0.5–64.6)	15	6.8 [3.4–12.0] (0.9–23.6)	11.3 [3.6–21.3] (2.0–28.9)	0.255	**0.003**	0.315	0.635
NK, %	25	8.0 [5.3–11.3] (1.8–15.2)	27.7 [20.2–29.8] (14.4–36.1)	15	4.0 [2.7–8.2] (1.0–26.0)	11.4 [8.1–18.3] (4.9–32.8)	**<0.001**	**<0.001**	**0.029**	**<0.001**
Tregs, %	25	2.7 [1.0–9.2] (0.2–45.4)	2.9 [1.6–8.8] (0.2–31.3)	15	0.4 [0.1–5.5] (0.01–16.7)	1.3 [0.7–5.0] (0.1–18.5)	0.419	**0.022**	**0.031**	0.072

*Wilcoxon-signed rank tests comparing changes in values after vs. before antigen stimulation. P<0.05 are bolded.

^†^Mann-Whitney U tests comparing values before or after antigen stimulation between healthy controls and participants with EP-NC. P<0.05 are bolded.

^‡^Not computed because all values were 0.

### Comparison of specific immunological parameters between participants with EP-NC before treatment and healthy controls

The specific inflammatory response between participants with EP-NC prior to treatment and healthy controls was significantly different in several parameters **(Table 2)**. Compared with participants with EP-NC, healthy controls had stronger responses to antigen stimulation for proinflammatory cytokines such as TNF-α (p<0.001) and IL-6 (p = 0.010), a higher response of the TH2 cytokine IL-5 (p = 0.019) and a lower response of the TH2 cytokine IL-4 (p = 0.031). Regarding cells, the percentage of NK cells was significantly lower in participants with EP-NC vs. healthy controls (p<0.001).

**Table 2:** Specific inflammatory response detected in the supernatant of PBMC *in vitro* stimulated with *T*. *solium* antigen comparing healthy controls and participants with EP-NC prior to treatment.

	Specific inflammatory response*, median [IQR] (min-max)		
	Healthy controls	Participants with EP-NC prior to treatment	p-value^†^
IL-1β (pg/mL)	11.3 [1.8–47.8] (-68.1–83.3)	0.7 [-19.8–46.0] (-1127.9–4022.4)	0.137
IL-17A (pg/mL)	0 [0–0] (0–4.2)	0 [0–0] (-35.8–28.6)	0.305
CCL5 (pg/mL)	30.6 [16.2–43.0] (-82.2–244.7)	9.7 [-41.4–145.9] (-326.6–1515.0)	0.472
IL-6 (pg/mL)	77.8 [26.3–218.9] (-117.3–361.6)	-17.1 [-139.6–108.5] (-3190.7–11382.5)	**0.010**
TNF-α (pg/mL)	2.8 [0.8–4.4] (-4.7–76.0)	0 [0–0] (0–141.9)	**<0.001**
IFN-γ (pg/mL)	0 [0–0] (0–8.4)	0 [0–0] (-6.9–250.7)	0.499
IL-4 (pg/mL)	0 [0–0] (0–0)	0 [0–0] (0–126.0)	**0.031**
IL-5 (pg/mL)	0.1 [0–0.5] (-0.2–4.1)	0 [0–0] (-37.7–9.0)	**0.019**
Proliferative, %	0.1 [-0.7–1.0] (-5.4–12.5)	2.8 [-0.6–9.7] (-5.0–72.4)	0.084
Naïve, %	-1.4 [-5.5–1.8] (-31.4–17.7)	0.9 [-1.0–4.5] (-21.8–25.4)	0.088
Central memory, %	0.1 [-1.4–2.7] (-17.9–27.7)	1.3 [0–3.1] (-20.3–9.3)	0.635
Effector memory, %	0.2 [-0.3–0.7] (-12.5–18.5)	1.3 [0.2–2.9] (-16.5–7.4)	0.199
Bregs, %	0.3 [-0.1–5.8] (-1.1–14.8)	1.0 [0.2–2.9] (-1.1–9.1)	0.655
NKT, %	0.7 [-0.8–3.8] (-13.1–13.0)	2.1 [0.7–7.0] (-1.6–10.3)	0.094
NK, %	17.3 [13.5–21.8] (9.3–26.3)	6.7 [4.5–11.2] (1.9–12.6)	**<0.001**
Tregs, %	0.3 [-0.4–1.0] (-14.1–7.2)	0.9 [0.1–1.0] (-4.0–7.4)	0.199

*Difference in parameter after minus before Ag stimulation, based on values in Table 1.

^**†**^Mann-Whitney U tests comparing specific inflammatory responses between healthy controls and participants with EP-NC. p<0.05 are bolded.

### Changes in specific immunological parameters among participants with EP-NC after treatment

**Table 3** shows changes in the specific inflammatory responses among participants with EP-NC from pre-treatment to 1-month post-treatment and 6-months post-treatment, and between 1-month post-treatment to 6-months post-treatment. No statistically significant changes were detected in cytokine levels. A significant decrease in the proliferative response was observed from before treatment to 1 month after.

**Table 3:** Changes in specific inflammatory response among participants with EP-NC before and 1- and 6-months after treatment.

	Pre-treatment to Month 1 post-treatment		Month 1 to Month 6		Pre-treatment to Month 6 post-treatment	
	Change in specific inflammatory response, median [IQR] (min-max)	p-value*	Change in specific inflammatory response, median [IQR] (min-max)	p-value*	Change in specific inflammatory response, median [IQR] (min-max)	p-value*
IL-1β, pg/mL	-1.5 [-132.8–78.0] (-3594.9–462.4)	0.646	5.5 [-18.1–75.3] (-1126.0–434.8)	0.435	3.0 [-65.3–32.6] (-4051.8–1217.2)	0.632
IL-17A, pg/mL	0 [0–2.4] (-10.0–36.2)	0.153	0 [-2.4–0] (-311.2–168.2)	0.626	0 [-1.8–2.1] (-311.2–172.5)	0.799
CCL5, pg/mL	11.6 [-121.7–110.6] (-1740.0–321.6)	0.965	4.4 [-27.5–182.7] (-195.3–574.7)	0.154	64.4 [-46.9–217.0] (-1406.0–625.5)	0.117
IL-6, pg/mL	62.7 [-68.0–232.4] (-13158.9–3158.7)	0.141	19.8 [-199.0–428.1] (-3128.5–6545.1)	0.409	127.2 [-130.3–588.0] (-11170.7–6477.0)	0.174
TNF-α, pg/mL	0 [0–1.4] (-141.9–7.2)	0.297	0 [-1.3–0] (-7.2–6.4)	0.313	0 [0–0] (-141.9–6.4)	0.438
IFN-γ, pg/mL	0 [0–2] (-247.8–50.0)	0.502	0 [-1.5–0] (-636.3–3784.7)	0.787	0 [0–11.2] (-621.6–3610.5)	0.610
IL-4, pg/mL	0 [0–0] (-126.0–455.4)	0.700	0 [0–0] (-455.4–41.8)	0.470	0 [0–0] (-118.6–41.8)	0.846
IL-5, pg/mL	0 [0–0] (-40.0–37.7)	0.734	0 [0–2.1] (-6.6–63.4)	0.055	0 [0–0] (-8.5–63.4)	0.203
Proliferative, %	**-2.5** **[-8.8–0.9]** **(-52.8**–**14.9)**	**0.001**	0 [-3.5–6.7] (-32.7–38.8)	0.771	-1.4 [-9.3–2.4] (-61.5–40.1)	0.109
Naïve, %	-0.3 [-3.0–2.7] (-22.4–13.4)	0.787	1.7 [-2.1–6.7] (-8.5–10.7)	0.424	0 [-4.3–4.6] (-23.1–24.1)	0.985
Central memory, %	-0.2 [-1.5–0.7] (-7.9–22.0)	0.982	-0.1 [-3.9–3.0] (-7.7–11.1)	0.791	-0.2 [-3.1–6.4] (-3.7–17.6)	0.733
Effector memory, %	-0.5 [-1.3–7.3] (-5.1–20.4)	0.622	-1.1 [-3.6–0.6] (-7.8–6.3)	0.301	-0.6 [-3.4–2.8] (-8.0–16.2)	0.791
Bregs, %	0.5 [-0.6–2.3] (-3.7–46.1)	0.305	-0.1 [-3.8–2.6] (-55.8–53.6)	0.733	0.1 [-1.4–1.0] (-9.7–53.9)	0.970
NKT, %	0.7 [-2.8–4.7] (-9.4–19.8)	0.636	-2.6 [-3.3–1.8] (-16.6–14.3)	0.311	1.1 [-3.3–3.4] (-26.0–11.5)	0.733
NK, %	-3.0 [-3.3–1.0] (-6.5–5.0)	0.340	2.9 [-0.7–8.2] (-2.5–14.1)	0.077	3.0 [0.5–10.1] (-6.1–14.1)	0.052
Tregs, %	-0.02 [-0.8–1.5] (-7.8–28.9)	0.839	-0.1 [-2.0–1.3] (-29.7–20.9)	0.850	-0.1 [-0.7–1.2] (-6.5–27.7)	0.910

*Wilcoxon-signed rank tests limited to individuals with available data at both time points. p<0.05 are bolded.

### Associations of pre-treatment specific inflammatory responses with percent reduction of cyst volume after treatment

As shown in **Table 4**, the immunological parameters evaluated were not significantly associated with treatment response, except IFNγ, which had a significant negative association with treatment response (p = 0.006). However, when removing a potential outlier with a very robust IFNγ response, but no treatment response, this negative association was attenuated and no longer statistically significant (p = 0.277).

**Table 4:** Associations of pre-treatment specific inflammatory response with percent reduction in cyst volume after treatment.

Specific inflammatory response	n	R-square	Beta estimate*	95% Confidence interval	p-value
IL-1β, pg/mL	42	0.03	0.004	-0.01–0.02	0.586
IL-17A, pg/mL	38	0.02	0.01	-1.22–1.24	0.986
CCL5, pg/mL	35	0.02	0.01	-0.03–0.05	0.574
IL-6, pg/mL	40	0.04	0.001	-0.004–0.006	0.647
TNF-α, pg/mL	34	0.05	0.20	-0.26–0.68	0.411
IFN-γ, pg/mL	**36**	**0.24**	**-0.27**	**-0.45**–**-0.08**	0.006*
IL-4, pg/mL	41	0.04	0.20	-0.30–0.71	0.417
IL-5, pg/mL	31	0.10	-0.54	-1.93–0.85	0.435
Proliferative, %	38	0.09	0.25	-0.40–0.91	0.447
Naïve, %	15	0.06	-0.04	-2.29–2.21	0.972
Central memory, %	15	0.07	-0.62	-3.80–2.54	0.674
Effector memory, %	15	0.24	2.34	-0.87–5.64	0.135
Bregs, %	15	0.16	3.73	-3.37–10.84	0.273
NKT, %	15	0.10	-1.97	-8.17–4.23	0.499
NK, %	15	0.14	3.13	-3.44–9.69	0.317
Tregs, %	15	0.06	0.37	-9.15–9.89	0.934

Multivariable linear regression models adjusted for age and pre-treatment parasite volume. p<0.05 are bolded.

*When excluding a potential outlier with a very robust IFN-γ response, but no treatment response: R-square: 0.08; beta estimate: -0.16; 95% confidence interval: -0.44–0.13; p-value: 0.277.

### Correlations between pre-treatment specific peripheral inflammatory response and pre-treatment CSF characteristics

As shown in **S1 Table**, some pre-treatment specific peripheral inflammatory responses were significantly correlated with pre-treatment CSF cells, protein, and glucose. Inflammatory pre-treatment CSF characteristics (increased cells and proteins and decreased glucose) were associated with higher level of pre-treatment TNF-α (significant with cells, p = 0.028), lower levels of pre-treatment IL-17 (significant with proteins, p = 0.001) and higher levels of both pre-treatment T regs and naïve cells (significant for proteins [p = 0.005 and p = 0.013, respectively] and glucose [p = 0.031 and p = 0.014, respectively]).

### Description of peripheral hormones among participants with EP-NC before and after treatment, and associations with treatment response and immunological parameters

There was a statistically significant reduction of estradiol from pre-treatment to 1-month post-treatment (p = 0.034) among males; however, there were no other statistically significant changes in peripheral hormones over time (**Table 5**). Among males, there was a trend towards increased levels of testosterone comparing pretreatment with 6 months posttreatment (p = 0.064). Associations of pre-treatment hormones with treatment response were not statistically significant (**Table 6**).

**Table 5:** Peripheral hormone levels among participants with EP-NC stratified by sex at pre-treatment, 1-month post-treatment, and 6-months post-treatment.

Hormone		Pre-treatment, median [IQR] (min-max)		1-month post-treatment, median [IQR] (min-max)		6 months post-treatment, median [IQR] (min-max)		Pre-treatment to Month 1 post-treatment		Month 1 to Month 6		Pre-treatment to Month 6 post-treatment	
								Difference, median [IQR] (min-max)	p-value*	Difference, median [IQR] (min-max)	p-value*	Difference, median [IQR] (min-max)	p-value*
**Females**													
Estradiol, pg/mL	Pre-menopause	7	41.1 [19.5–106.0] (14.4–229.2)	7	29.6 [13.7–62.4] (9.2–111.1)	5	30.6 [28.2–33.2] (18.7–69.0)	-11.5 [-92.3–48.0] (-196.5–86.2)	0.469	19.5 [-3.4–21.4] (-34.2–36.3)	0.625	-28.1 [-72.8–11.1] (-160.2–13.8)	0.313
	Post-menopause	6	16.9 [10.3–20.5] (2.2–20.9)	6	10.6 [9.1–15.3] (9.0–15.5)	6	13.5 [10.8–17.2] (9.7–43.1)	-5.8 [-9.4–-0.1] (-9.5–13.1)	0.438	1.7 [-0.5–3.3] (-2.5–34.2)	0.313	-2.1 [-4.6–10.6] (-6.2–24.8)	1.000
Testosterone, pg/mL		NA	NA	NA	NA	NA	NA	NA	NA	NA	NA	NA	NA
Cortisol, mg/dL		13	325.9 [160.3–397.4] (31.3–1075.5)	12	223.4 [124.2–306.8] (9.7–703.0)	12	278.6 [222.7–427.1] (5.3–511.5)	-73.0 [-294.2–117.5] (-760.7–375.7)	0.569	-19.3 [-146.1–210.0] (-288.0–368.2)	0.831	45.9 [-126.5–104.8] (-906.8–464.6)	0.970
Prolactin, mcg/L		13	11.6 [6.2–14.7] (3.2–24.1)	13	9.0 [6.6–11.5] (5.0–17.8)	13	10.0 [8.5–16.2] (4.4–104.6)	-2.3 [-5.0–3.1] (-10.8–6.4)	0.414	1.2 [-1.5–6.7] (-8.9–99.4)	0.305	2.6 [-3.8–4.3] (-19.7–89.9)	0.414
**Males**													
Estradiol, pg/mL		12	12.2 [9.4–20.9] (5.4–30.6)	13	9.6 [8.3–13.1] (5.9–17.2)	12	11.1 [8.9–19.5] (6.8–27.6)	-**3.8 [-10.6–-0.7]** **(-16.0**–**5.8)**	**0.034**	2.6 [-0.6–7.3] (-4.8–17.5)	0.110	0.2 [-3.9–1.4] (-20.4–13.1)	0.700
Testosterone, pg/mL		14	5.4 [3.5–6.6] (0.2–16.5)	13	5.6 [3.5–8.0] (0.2–12.4)	12	7.3 [5.3–9.0] (0.2–12.4)	0.4 [-2.8–1.5] (-6.8–11.5)	0.850	0.9 [-0.3–6.9] (-12.2–8.9)	0.413	2.4 [0.2–2.5] (-15.7–8.1)	0.064
Cortisol, mg/dL		12	379.3 [146.5–501.1] (113.7–756.5)	11	391.7 [238.5–504.7] (8.6–930.4)	11	318.6 [168.3–492.8] (106.0–986.8)	33.8 [-193.7–310.5] (-401.4–394.3)	0.577	-40.1 [-167.0–155.0] (-658.3–816.6)	0.520	-39.9 [-246.3–250.2] (-263.9–873.1)	0.700
Prolactin, mcg/L		12	7.0 [4.9–13.8] (3.2–14.6)	11	8.2 [3.5–11.6] (1.7–12.5)	12	5.8 [5.2–9.6] (3.8–18.3)	-0.02 [-2.5–2.7] (-12.8–4.9)	1.000	1.1 [-3.0–4.0] (-5.9–6.7)	0.577	-0.2 [-5.4–1.5] (10.0–9.4)	0.733

NA, not applicable. *Wilcoxon-signed rank tests comparing changes in hormone levels at two time points. p<0.05 are bolded.

**Table 6:** Association of pre-treatment peripheral hormones with percent reduction in cyst volume after treatment.

Hormone	n	R-Square	Beta estimate	95% Confidence interval	p-value
Estradiol, pg/mL	25	0.23	0.02	-0.26–0.33	0.890
Testosterone, pg/mL	14	0.05	-0.73	-5.18–3.72	0.725
Cortisol, mg/dL	25	0.33	0.04	-0.01–0.08	0.135
Prolactin, mcg/L	25	0.25	-0.18	-2.33–1.97	0.861

Multivariable linear regression models adjusted for age, sex, and pre-treatment parasite volume. The model for testosterone was not adjusted for sex as testosterone was only measured among males.

Correlations between endocrine parameters and specific inflammatory responses, both among participants with EP-NC prior to treatment, are shown in **S2 Table**. Cortisol was negatively correlated with IL-6 (Spearman correlation coefficient -0.44; p = 0.035) and positively correlated with IFN-γ (Spearman correlation coefficient +0.51; p = 0.022).

## Discussion

EP-NC is the most severe form of NC. The localization of parasites in this compartment favors an inflammatory phenomenon which, sooner or later in the course of the infection, can be life threatening. Estimating the time from infection to diagnosis has been possible by studying patients living in a non-endemic country who became infected during a temporary stay in an endemic country and individuals with NC migrating from endemic to non-endemic countries. A long latency period that can last up to 20 years has been observed [16,17], demonstrating the chronicity of this form of NC and highlighting the potential dangers of the central inflammation associated with this disease [18]. Central inflammation can be exacerbated by anthelmintic treatment. For this reason, high doses of corticosteroids are used to control it, which might reduce the effectiveness of the anthelmintic treatment in destroying the parasite [6]. This scenario points to the relevance and the complexity of the inflammatory response and its treatment in the severity and evolution of NC. This study was designed to describe the characteristics of peripheral immune and endocrine systems in this severe presentation of NC and their possible association with response to treatment. As communication between central and peripheral compartments exists [19,20], we also sought to evaluate if the specific peripheral inflammatory response reflects the central inflammation present in these patients.

We observed an anti-inflammatory/low-responsiveness immunity to cysticercal antigens in EP-NC that may contribute to the chronicity of the infection. PBMC stimulation by specific antigen mainly increased immunological parameters of TH2 and regulatory responses (significant increases of IL-4, Bregs and Tregs cells). Also compared with healthy controls, participants with EP-NC prior to treatment had a significantly weaker specific response to antigen among proinflammatory cytokines (IL-6 and TNF-α) and NK cells. In fact, the response of proinflammatory cytokines among participants with EP-NC did not reach statistical significance when compared with the no-antigen stimulation condition. Participants with EP-NC had a statistically significant IL-4 response, which was absent among healthy controls. Lymphocytes proliferate after specific stimulation, but this was not significantly different from proliferation among healthy controls, and much lower than proliferation among patients with asymptomatic NC [15].

These results are in accordance with a previous study comparing peripheral inflammatory response in healthy controls, patients with EP-NC, and patients with parenchymal NC [21]. In this study, the authors found the presence of an anti-inflammatory response in EP-NC, while in parenchymal disease a peripheral pro-inflammatory response was mainly observed [21]. These findings are of great relevance as the two different inflammatory responses might be related to the differences in response to treatment observed between the 2 forms of the disease.

The presence of a regulatory response promoted by the parasite has been previously described among patients with NC [22,23]. The present study consolidates these previous findings by showing low T-cell responsiveness that could be promoted by a regulatory environment with increased Tregs and Bregs, as well as alternatively activated macrophages as previously reported in experimental infections [24]. In this context, the significant positive association between CSF inflammatory features (increased protein and decreased glucose) and peripheral T regs levels before treatment is interesting. Indeed, this observation reinforces the evidence that immunological regulatory mechanisms are promoted to facilitate parasite survival by controlling parasite-related central inflammation [22,25].

Another interesting observation in our study is the significant increase of NK cells after specific stimulation more importantly in healthy controls than in participants with EP-NC, which may imply a greater inflammatory potential of the NK cells from healthy controls to activate and respond to an eventual challenge. NK cells and the more recently identified cell population NK dendritic cells (NKDC) have been found to proliferate *in vitro* under inflammatory conditions (IL-18 and TLR agonists) [26]. On the other hand, the increase of NKT cells in participants with EP-NC (p = 0.094) may favor a TH2 response, as has been observed in other helminthiases by producing IL-4 [27]. The role of these two types of cells should be thoroughly studied in future research, but our results are consistent with a restricted state of immunoinflammatory response among patients with NC vs. healthy controls.

Anthelmintic treatment among participants with EP-NC did not result in significant peripheral immunological changes. This treatment is associated with a strong inflammatory reaction in the CNS with potentially dramatic clinical consequences [28–30], but this did not appear to have any major impact in the periphery. The concomitant administration of systemic corticosteroids with albendazole could explain the absence of changes in the peripheral immunity. It is indeed known that corticosteroids inhibit lymphocyte binding to endothelial cells, increase apoptosis of T and B cells in the thymus and the periphery, and inhibit TLR pathways in macrophages and DCs via direct suppression of p38/MAPK phosphorylation, which results in down-regulation of DC migration and maturation [25]. The concomitant use of corticosteroids has been demonstrated to reduce mortality and improve patient outcomes and is recommended by current treatment guidelines [12,31]. Although the positive effects of corticosteroids on the control of neuroinflammation are clear, the consequences of their effects on peripheral T cells should be examined in depth. Corticosteroids may prevent exacerbation of inflammatory signals in the CNS but may also promote chronicity of infection and poor response to treatment. New anti-inflammatory alternatives with less systemic effects could eventually be considered to improve the outcome of patients with EP-NC.

The lack of peripheral pre-treatment immunological parameters associated with treatment response is also likely related to this low responsiveness. The only cytokine significantly associated with treatment response was IFN-γ, but this cytokine had a very small concentration, and the association was not robust.

Regarding endocrine status, we found a significant reduction in estradiol among males 1 month after treatment. We have previously shown that estradiol was decreased in male patients with NC compared with controls without NC [8]. This observation could be a consequence of decreased aromatization of testosterone to estradiol by the enzyme aromatase P-450, with testicular aromatization being influenced by different cytokines and sex steroids [32]. Participants with EP-NC showed weaker responses than healthy controls in proinflammatory cytokines (IL-1β, CCL5, IL-6, TNF-α). Cytokines, such as IL-6 and TNF-α, have an important role in regulating estrogen synthesis in peripheral tissues, including normal and malignant breast tissues [33].

We found no significant association between endocrine status and response to treatment, and regarding the associations between endocrine and immunologic parameters, cortisol levels were negatively correlated with IL-6 (p = 0.035) and positively correlated with IFN-γ (p = 0.022). Measuring cortisol in serum is influenced by the circumstances around the sampling procedure that generally place stress on the patient and therefore may mask differences caused by the treatment. Further studies with larger sample sizes are needed to expand the knowledge of this topic.

In conclusion, our results suggest the presence of a depressed T lymphocyte response in EP-NC that could favor the chronicity of infection and suboptimal response to anthelmintic treatment. Further studies are required to identify the underlying mechanisms involved in this weakened peripheral immunity.

## Supporting information

S1 Fig Strategy for the selection of lymphocyte populations in peripheral blood from a patient stimulated *in vitro* with *T*. *solium* antigen. T cells (Naïve, central memory, effector cells), NKT/NK, B regs and T Regs were identified first by gating on the lymphocyte population using forward and side scatter parameters. Subsequently, the proliferated cells were selected and from them all the populations of T cells were identified.
(PDF)

S1 Table Correlation between pre-treatment cerebrospinal fluid cells, protein, and glucose with specific inflammatory response among participants with EP-NC prior to treatment. (DOCX)

S2 Table Correlation between pre-treatment peripheral hormones and specific inflammatory response among participants with EP-NC. (DOCX)
