# Supplementary figures and images for: Low responsiveness of peripheral lymphocytes in extraparenchymal neurocysticercosis
Source: PLoS Negl Trop Dis. 2023 Jun 1;17(6):e0011386. doi: 10.1371/journal.pntd.0011386 (PMC10263342; doi:10.1371/journal.pntd.0011386)

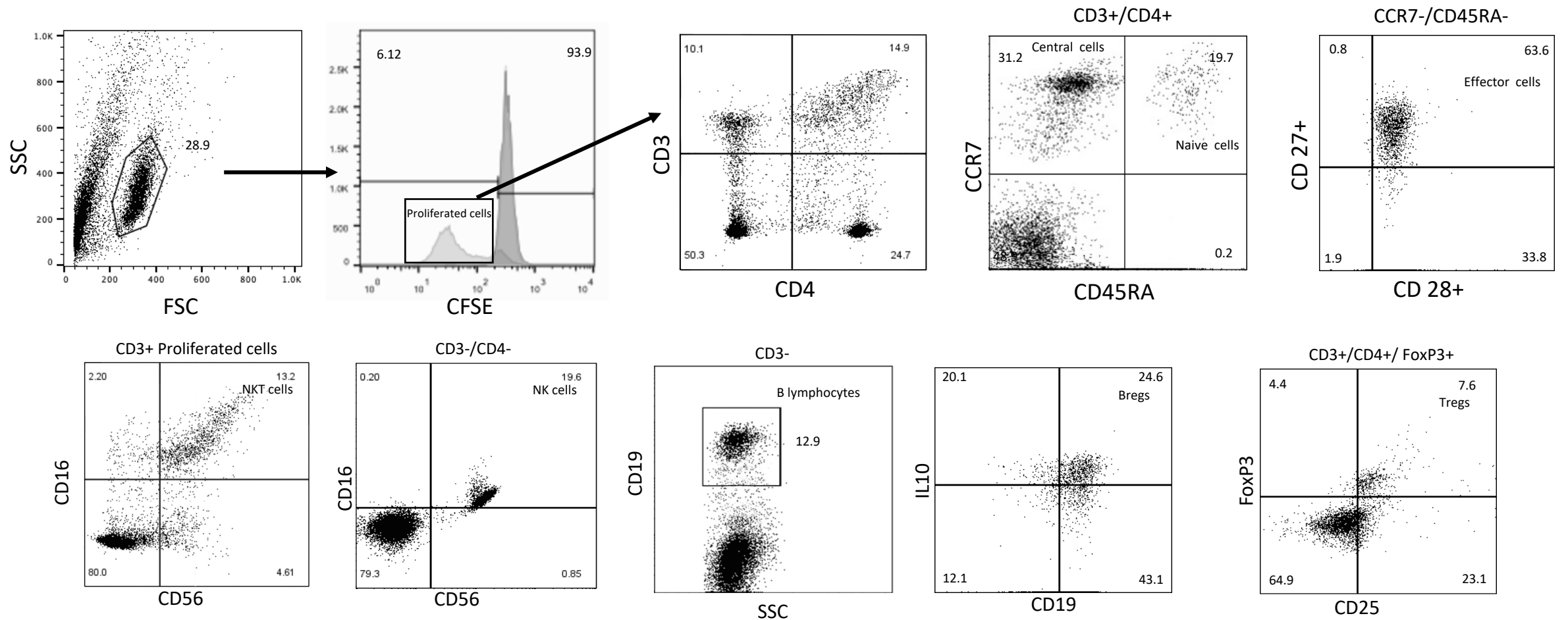

Supplement: S1 Fig — T cells (Naïve, central memory, effector cells), NKT/NK, B regs and T Regs were identified first by gating on the lymphocyte population using forward and side scatter parameters. Subsequently, the proliferated cells were selected and from them all the populations of T cells were identified. (PDF) [file pntd.0011386.s001.pdf]
