# Supplementary material for: Low responsiveness of peripheral lymphocytes in extraparenchymal neurocysticercosis
Source: PLoS Negl Trop Dis. 2023 Jun 1;17(6):e0011386. doi: 10.1371/journal.pntd.0011386 (PMC10263342; doi:10.1371/journal.pntd.0011386)
Supplement: S1 Table — (DOCX) [file pntd.0011386.s002.docx]

**S1 Table. Correlation between pre-treatment peripheral hormones and specific inflammatory response among participants with EP-NC**

|  | **Estradiol** | **Testosterone** | **Cortisol** | **Prolactin** |
| --- | --- | --- | --- | --- |
| **IL-1β** | 0.20 (n=25), 0.326 | -0.07 (n=14), 0.816 | -0.37 (n=25), 0.068 | 0.26 (n=25), 0.215 |
| **IL-17A** | 0.01 (n=21), 0.967 | 0.30 (n=11), 0.370 | -0.06 (n=22), 0.777 | 0.06 (n=22), 0.785 |
| **CCL5** | -0.27 (n=18), 0.284 | 0.18 (n=9), 0.637 | 0.32 (n=19), 0.185 | 0.04 (n=19), 0.881 |
| **IL-6** | -0.17 (n=24), 0.439 | 0.13 (n=13), 0.681 | **-0.44 (n=23), 0.035** | 0.14 (n=23), 0.513 |
| **TNF-α** | 0.02 (n=18), 0.947 | -0.03 (n=10), 0.943 | -0.26 (n=18), 0.303 | 0.26 (n=18), 0.303 |
| **IL-4** | -0.04 (n=25), 0.846 | -0.08 (n=14), 0.795 | 0.26 (n=25), 0.215 | -0.14 (n=25), 0.491 |
| **IL-5** | 0.26 (n=18), 0.303 | * (n=10) | -0.04 (n=19), 0.861 | 0.00 (n=19), 1.000 |
| **IFN-γ** | -0.29 (n=19), 0.241 | -0.51 (n=11), 0.107 | **0.51 (n=20), 0.022** | -0.14 (n=20), 0.567 |
| **% Proliferative** | -0.08 (n=23), 0.720 | -0.35 (n=13), 0.242 | 0.23 (n=23), 0.295 | -0.11 (n=23), 0.620 |
| **% Naïve** | -0.30 (n=5), 0.624 | -0.50 (n=3), 0.667 | 0.20 (n=5), 0.747 | 0.30 (n=5), 0.624 |
| **% Central memory** | 0.60 (n=5), 0.285 | 0.50 (n=3), 0.667 | -0.10 (n=5), 0.873 | -0.10 (n=5), 0.873 |
| **% Effector memory** | **-1.00 (n=5), <0.001** | -0.50 (n=3), 0.667 | 0.30 (n=5), 0.624 | 0.00 (n=5), 1.000 |
| **% Bregs** | -0.60 (n=5), 0.285 | 0.50 (n=3), 0.667 | -0.50 (n=5), 0.391 | 0.60 (n=5), 0.285 |
| **% NKT** | -0.50 (n=5), 0.391 | -0.50 (n=3), 0.667 | -0.30 (n=5), 0.624 | 0.00 (n=5), 1.000 |
| **% NK** | 0.20 (n=5), 0.747 | -0.50 (n=3), 0.667 | -0.70 (n=5), 0.188 | 0.80 (n=5), 0.104 |
| **% Tregs** | -0.67 (n=5), 0.219 | 0.50 (n=3), 0.667 | 0.88 (n=5), 0.054 | -0.36 (n=5), 0.553 |

Spearman correlation coefficient (number of participants), and p-value are presented, limited to individuals with data on both parameters. p<0.05 are bolded. *Unable to compute correlation coefficient because all values of IL-5 specific response were 0. ^†^Unable to assess statistical significance.
