# Supplementary material for: Low responsiveness of peripheral lymphocytes in extraparenchymal neurocysticercosis
Source: PLoS Negl Trop Dis. 2023 Jun 1;17(6):e0011386. doi: 10.1371/journal.pntd.0011386 (PMC10263342; doi:10.1371/journal.pntd.0011386)
Supplement: S2 Table — (DOCX) [file pntd.0011386.s003.docx]

**S2 Table. Correlation between pre-treatment cerebrospinal fluid cells, protein, and glucose with specific inflammatory response among participants with EP-NC prior to treatment**

|  | **Pre-treatment CSF cells** | **Pre-treatment CSF protein** | **Pre-treatment CSF glucose** |
| --- | --- | --- | --- |
| **IL-1β** | 0.04 (n=38), 0.793 | -0.08 (n=36), 0.662 | -0.25 (n=38), 0.130 |
| **IL-17A** | -0.29 (n=34), 0.092 | **-0.54 (n=32), 0.001** | 0.31 (n=34), 0.079 |
| **CCL5** | 0.31 (n=33), 0.077 | 0.21 (n=31), 0.248 | -0.16 (n=33), 0.388 |
| **IL-6** | 0.11 (n=36), 0.535 | 0.11 (n=34), 0.519 | -0.22 (n=36), 0.193 |
| **TNF-α** | **0.40 (n=30), 0.028** | 0.29 (n=28), 0.136 | -0.25 (n=30), 0.191 |
| **IL-4** | -0.10 (n=37), 0.561 | -0.19 (n=27), 0.276 | 0.21 (n=37), 0.217 |
| **IL-5** | 0.21 (n=29), 0.273 | 0.148 (n=27), 0.463 | -0.12 (n=29), 0.551 |
| **IFN-γ** | 0.08 (n=33), 0.653 | -0.08 (n=31), 0.683 | -0.27 (n=33), 0.123 |
| **% Proliferative** | -0.02 (n=35), 0.889 | -0.12 (n=33), 0.510 | -0.20 (n=35), 0.246 |
| **% Naïve** | 0.002 (n=14), 0.994 | **0.69 (n=12), 0.013** | **-0.64 (n=14), 0.014** |
| **% Central memory** | 0.22 (n=14), 0.444 | -0.10 (n=12), 0.762 | 0.11 (n=14), 0.707 |
| **% Effector memory** | -0.43 (n=14), 0.123 | -0.21 (n=12), 0.513 | 0.07 (n=14), 0.805 |
| **% Bregs** | 0.02 (n=14), 0.940 | 0.43 (n=12), 0.159 | 0.02 (n=14), 0.958 |
| **% NKT** | 0.02 (n=14), 0.940 | 0.26 (n=12), 0.417 | -0.25 (n=14), 0.390 |
| **% NK** | -0.09 (n=14), 0.765 | 0.12 (n=12), 0.713 | -0.05 (n=14), 0.863 |
| **% Tregs** | 0.41 (n=14), 0.142 | **0.75 (n=12), 0.005** | **-0.57 (n=14), 0.031** |

Spearman correlation coefficient (number of participants), and p-value are presented, limited to individuals with data on both parameters. p<0.05 are bolded.
